# Imaging of kiss-and-run exocytosis of surface receptors in neuronal cultures

**DOI:** 10.3389/fncel.2014.00363

**Published:** 2014-11-03

**Authors:** Cristina Roman-Vendrell, Michael Chevalier, Agnes M. Acevedo-Canabal, Francheska Delgado-Peraza, Jacqueline Flores-Otero, Guillermo A. Yudowski

**Affiliations:** ^1^Institute of Neurobiology, University of Puerto Rico Medical Sciences CampusSan Juan, PR, USA; ^2^Department of Physiology, School of Medicine, University of Puerto RicoSan Juan, PR, USA; ^3^Department of Biochemistry and Biophysics, California Institute for Quantitative Biosciences, University of California San FranciscoSan Francisco, CA, USA; ^4^Department of Anatomy and Neurobiology, School of Medicine, University of Puerto RicoSan Juan, PR, USA

**Keywords:** exocytosis, TIRF microscopy, neurons, receptors, cell surface, GPCR, AMPA receptors

## Abstract

Transmembrane proteins are continuously shuttled from the endosomal compartment to the neuronal plasma membrane by highly regulated and complex trafficking steps. These events are involved in many homeostatic and physiological processes such as neuronal growth, signaling, learning and memory among others. We have previously shown that endosomal exocytosis of the B2 adrenergic receptor (B2AR) and the GluR1-containing AMPA receptor to the neuronal plasma membrane is mediated by two different types of vesicular fusion. A rapid type of exocytosis in which receptors are delivered to the plasma membrane in a single kinetic step, and a persistent mode in which receptors remain clustered at the insertion site for a variable period of time before delivery to the cell surface. Here, by comparing the exocytosis of multiple receptors in dissociated hippocampal and striatal cultures, we show that persistent events are a general mechanism of vesicular delivery. Persistent events were only observed after 10 days *in vitro*, and their frequency increased with use of the calcium ionophore A23187 and with depolarization induced by KCl. Finally, we determined that vesicles producing persistent events remain at the plasma membrane, closing and reopening their fusion pore for a consecutive release of cargo in a mechanism reminiscent of synaptic kiss-and-run. These results indicate that the delivery of transmembrane receptors to the cell surface can be dynamically regulated by kiss-and-run exocytosis.

## Introduction

Cellular sensitivity to external stimuli is tightly controlled by the number and location of transmembrane receptors at the plasma membrane. Receptors are removed and delivered to the cell surface by endocytosis and exocytosis (Collingridge et al., 2004; von Zastrow and Williams, 2012). Both events are ubiquitous and fundamental in many biological processes such as learning and the development of tolerance (Bredt and Nicoll, 2003; Martini and Whistler, 2007; Koch and Höllt, 2008; Flores-Otero et al., 2014).

Although synaptic exocytosis has been extensively characterized, less is known about the mechanisms and kinetics of receptor exocytosis to the neuronal plasma membrane (De Camilli and Jahn, 1990; Maletic-Savatic and Malinow, 1998; Maletic-Savatic et al., 1998; Stevens and Williams, 2000; Jaiswal et al., 2009). Calcium-evoked somatic and dendritic exocytosis (CEDE) was initially described in the 1990's using time-lapse microscopy and capacitance measurements (Huang and Neher, 1996; Maletic-Savatic and Malinow, 1998; Maletic-Savatic et al., 1998). In those studies, release of FM dyes from intracellular stores and changes in capacitance were used to investigate the fundamental mechanism underlying non-synaptic exocytosis from neurons. This work suggested that receptors could be delivered by exocytosis, although direct visualization was not described until much later (Kopec et al., 2006; Yudowski et al., 2006; Kennedy and Ehlers, 2011).

We previously used total internal reflection fluorescence (TIRF) microscopy to directly visualize the exocytosis of the B2 adrenergic receptor (B2AR), the mu opioid receptor (MOR), and the GluR1-containing AMPA-type receptor in dissociated neuronal cultures with high temporal resolution (Yudowski and von Zastrow, 2011; Roman-Vendrell and Yudowski, 2015). During these studies we identified transient and *persistent* modes of exocytosis. In persistent events, receptors were retained at their site of insertion for a variable period of time before their disappearance (Yudowski et al., 2006, 2007; Yu et al., 2010; Roman-Vendrell et al., 2012). Persistent events were only observed in neuronal cultures expressing SEP-B2AR and SEP-GluR1, but not SEP-MOR. Interestingly, specific amino acid sequences in the receptor carboxy tail can target receptors to persistent events (Yu et al., 2010). Ehlers' group utilized a dual label transferrin receptor (TfR) to investigate receptor exocytosis in dendritic spines, but failed to observe persistent events in these structures (Kennedy et al., 2010). More recently, a different group detected the same events in neuronal cultures. Using rapid changes in extracellular pH, Jullié et al. proposed that persistent events are originated from recycling endosomes and their behavior at the plasma membrane could be explained by fusion pore closure similar to kiss-and-run (Jullié et al., 2014).

Here we sought to investigate persistent events further. We analyzed and compared the exocytosis of the TfR the B2AR and the GluR1 in hippocampal and striatal cultures. Persistent events were observed only in neurons >10 days *in vitro* (DIV), their retention at the plasma membrane was not mediated by the endocytic machinery and their frequency was increased by depolarization and cytoplasmic calcium. Interestingly, a fraction of persistent events were followed by a second exocytotic event at the same location, creating a hot-spot of receptor exocytosis. Our results suggest that persistent events are exocytotic vesicles that partially retain their cargo after an initial release. These vesicles close and reacidify remaining at the plasma membrane for consecutive exocytic events, in a mechanism reminiscent of synaptic kiss-and-run exocytosis.

## Results

**Figure 1:**
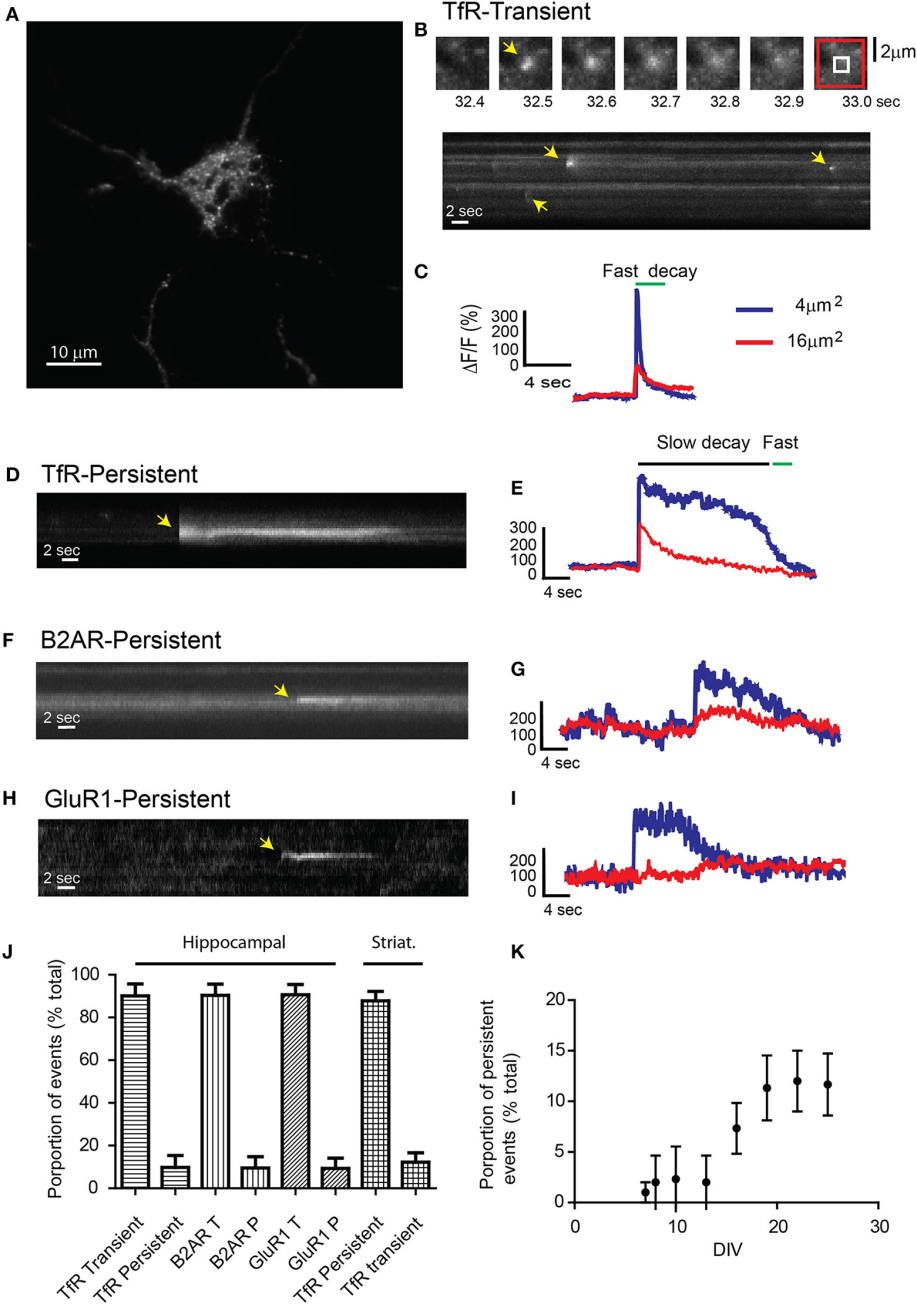
**Receptor exocytosis is mediated by two different modes of vesicular fusion**. **(A)** Dissociated hippocampal cultures (>15 DIV) were transfected with TfR-SEP and imaged under TIRF illumination. **(B)** Sequential images from the cell in **(A)** show a single transient exocytotic event. Red and white boxes define a 4 and 16 μm^2^ regions of interest analyzed in **(C)** (Top). Kymograph obtained from **(A)** shows multiple exocytic events (yellow arrows). **(C)** Maximum intensity measurements from **(A)** depict a rapid increase indicating fusion pore opening and a subsequent decay, indicating cargo release to the plasma membrane. **(D)** Kymograph depicting a single TfR-SEP persistent event in which receptors remain clustered at their site of insertion. Yellow arrow indicates exocytic event. **(E)** Maximum intensity measurements from **(D)** show fusion pore opening and lateral receptor diffusion from the insertion site. **(F)** Kymograph of a representative SEP-B2AR persistent event. **(G)** Intensity measurements from **(F)**. **(H)** SEP-GluR1 persistent insertion event. **(I)** Kymograph from **(H)**. **(J)** Proportion of transient vs. persistent events was counted for all the receptors in hippocampal cultures and for TfR-SEP in hippocampal and striatal cultures. Persistent events were 9.8 ± 5.5% from the total of TfR events. B2AR showed 8.5 ± 5.1% and Glur1 7.3 ± 4.8% (*n* = 7–10 cells) **(K)** Proportion of TfR-SEP persistent events during neuronal development *in vitro*.

We used live-cell TIRF microscopy to investigate the dynamics of receptor exocytosis in neuronal cultures. We utilized TfRs tagged with the pH-sensitive GFP phluorin on the extracellular domain to increase their visibility at the plasma membrane (Miesenbock et al., 1998; Sankaranarayanan et al., 2000; Yudowski et al., 2006; Araki et al., 2010). TfRs are ideal because they are constitutively endocytosed and recycled from the endosomal compartment in heterologous systems and hippocampal neurons (Cameron et al., 1991; Burack et al., 2000; Park et al., 2004). Hippocampal cultures transfected with TfR-SEP were imaged by TIRF microscopy at 10 Hz in a controlled environment. To avoid imaging non-neuronal cells, transfected neurons were first morphologically identified under epifluorescence light before switching to TIRF illumination (Yudowski and von Zastrow, 2011). With TIRF microscopy, individual exocytotic events were easily observed as rapid discrete increases in fluorescence intensity (Figures 1A,B) (Supplemental Movie 1). Individual events can be observed in kymographs in which a cross section of the cell is depicted over time (Figure 1B bottom). Maximum intensity measurements over time from these events show a rapid increase in intensity (<0.3 s), which indicates the fusion of the exocytotic vesicle with the plasma membrane and a rapid exponential decay (~2 s) indicating receptor release and diffusion onto the plasma membrane (Figure 1C) (Jaiswal and Simon, 2007; Wennmalm and Simon, 2007). Intensity measurements from the surrounding plasma membrane confirmed the exocytosis of receptors by showing a small but sustained increase in fluorescence (Figures 1B,C, red trace).

Unexpectedly, for passive cargo, a second type of fusion event in which receptors remained clustered at the cell surface for a variable period (>4 s) before their disappearance was observed. In this case, opening of the fusion pore showed kinetics identical to those of the rapid events, but the fluorescence decay was significantly slower and not related to bleaching (Figures 1D,E and Supplemental Figure 1). These events showed similar kinetics and fluorescence intensity as those observed with SEP-B2AR and SEP-GluR1 (Figures 1F–I) (Yudowski et al., 2006, 2007). Persistent events were observed in similar proportion during our 1-min movies in hippocampal and striatal neurons (TfR, 9.8 ± 5.5%; B2AR, 8.5 ± 5.1%; GluR1, 7.3 ± 4.8%; TfR striatum, 12.2 ± 4.4%; *n* = 7–10 cells; Figure 1J) and were observed only in cultures after 10 DIV (Figure 1K) and not in heterologous systems.

**Figure 2:**
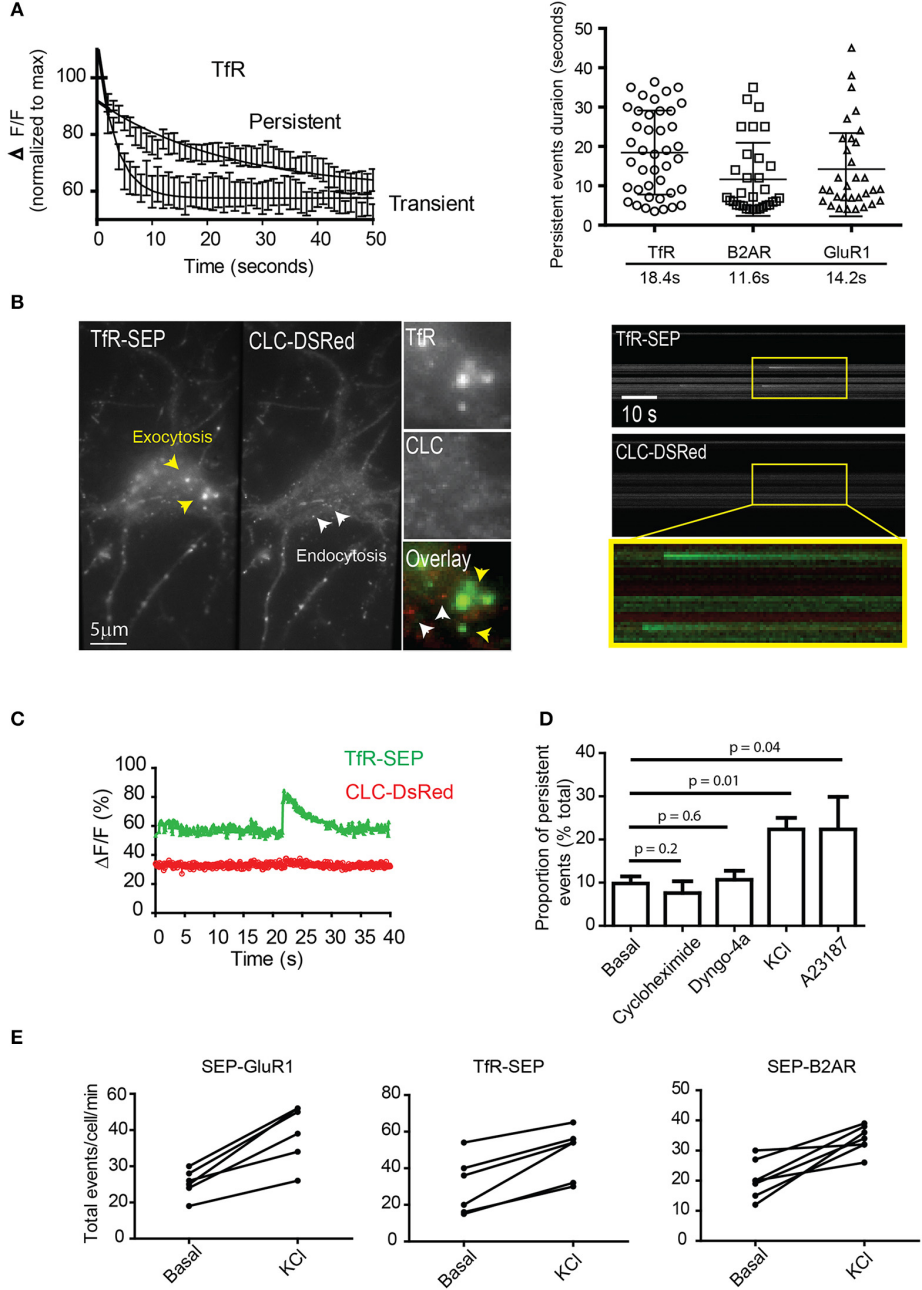
**Characterization of persistent events. (A)** Fluorescence intensity profiles from transient and persistent events were normalized to their maximum (fusion pore opening) and fitted to a single exponential decay (*n* = 12–18 events). Dot plot graph indicates duration of individual persistent events and their half-lives (mean and standard deviation) **(B)** Simultaneous dual-color TIRF imaging was performed on hippocampal neurons co-transfected with TfR-SEP and the clathrin light chain tagged with DsRed (CLC-DsRed). A maximum intensity projection from a 1 min movie shows a single neuron co-expressing TfR-SEP and CLC-DsRed. Individual endocytic events are observed as small spots in the red channel (2 by 2 pixels, white arrowhead and insert). Exocytic events are observed as bright spots generally bigger and more diffuse than endocytic events as previously reported elsewhere (yellow arrowheads). Kymographs from dual-color TIRF movies show multiple TfR-SEP events (top) and no colocalization with clathrin pits (bottom and overlay). **(C)** Intensity measurements of TfR-SEP and CLC-DsRed from **(A)** at the site of persistent insertion. **(D)** The frequency of persistent events was counted under basal conditions and in the presence of cycloheximide (18 h 10 μg/ml), 30 μM dyngo-4a (30 min before imaging), after depolarization with 25 mM KCl by perfusion and after application of 1 μM A23187. **(E)** Total number of exocytic events per cell were also increased after depolarization with 25 μM KCl. Error bars represents standard deviation. *P* values were calculated by unpaired two-tailed *t*-tests.

We compared and analyzed the changes in fluorescence from the time of exocytosis for TfR transient and persistent events. Multiple fluorescence traces were normalized to their maximum (exocytosis) and fitted to a single exponential decay (Figure 2A). Dot plot indicates half-lives of TfR, B2AR, and GluR1 receptors (Figure 2A, right panel). Interestingly, their half-lives were not significantly different for different receptors, suggesting a common vesicular mechanism. Next, we tested the possibility that persistent events are exocytotic events that rapidly undergo endocytosis. We co-expressed TfR-SEP with clathrin light chain tagged with Ds-Red (CLC-DsRed) in hippocampal neurons and performed simultaneous dual-color TIRF (Figure 2B). Events did not colocalize with CLC-DsRed, with a Pearson's coefficient = 0.07–0.05 (*n* = 7 cells). This indicates that their retention at the plasma membrane was not mediated by clathrin coated pits (Figures 2B,C).

To characterize persistent events further, we analyzed their relative proportion to total events. First, we investigated if they were originated from the biosynthetic pathway by preincubation with the protein synthesis inhibitor cyclohexamide (6 h) and found no significant difference between controls and treated cells (Figure 2D). Second, we tested if dynamin physically constricts the plasma membrane to retain receptors at their site of insertion. We preincubated cells with a specific inhibitor of dynamin and observed no significantly difference in their proportion when compared to basal conditions (Figure 2D). Next, we tested if depolarization with 25 mM KCl and an increase in cytosolic calcium affected the proportion of TfR-SEP persistent events. Bath application of KCl immediately elicited exocytosis. The total number of exocytotic events was significantly increased, and the proportion of persistent events rose to ~22% of the total (Figure 2D). Acute application of the ionophore A23187 also increased the frequency of persistent events (Figure 2D). Finally, we counted the total number of exocytic events before and after depolarization. Results showed that depolarization by KCl significantly increased the total number of exocytic events observed with all receptors (*p* < 0.05) (Figure 2E). Interestingly, the decay kinetics of persistent events after treatments was not significantly different from those observed under basal conditions.

**Figure 3:**
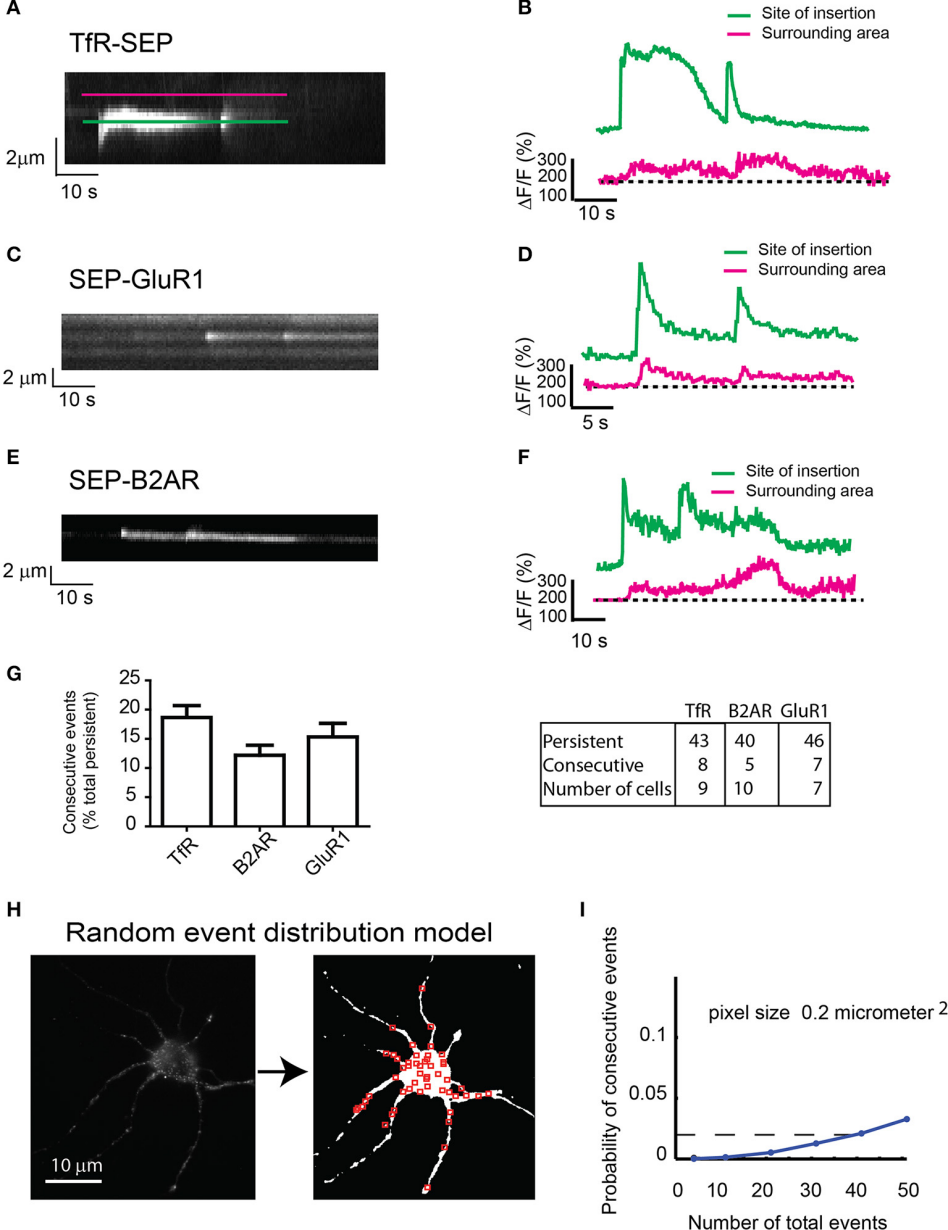
**Consecutive exocytosis of transmembrane receptors. (A)** Kymograph from hippocampal neurons transfected with TfR-SEP showing consecutive exocytotic events. **(B)** Maximum intensity measurements from **(A)**. Green or Blue line indicates intensity changes at the site of insertion; red line indicates lateral receptor spreading from the site of insertion along the plasma membrane. **(C)** Kymograph of SEP-GluR1 consecutive events in hippocampal neurons. **(D)** Intensity measurements from **(C)**. **(E)** Kymograph of SEP-B2AR consecutive events in hippocampal neurons. **(F)** Intensity measurements from **(E)**. **(G)** Number of persistent and consecutive events for all the receptors. **(H)** Conversion of a neuron to a threshold image in which red squares are randomly placed events (*n* = 51). **(I)** Probability of having one colocalized event as a function of total events based on the random distribution from **(H)**. The calculated probability of observing consecutive events is ~0.02.

During our imaging sessions, we noticed that some TfR-SEP persistent events were immediately followed by a second event at the same location (Figure 3A). Both events released cargo to the plasma membrane as measured by the net increase in basal fluorescence in areas surrounding the insertion site (red lines in Figure 3B). The fluorescence intensity between events rarely reached background levels, suggesting that the initial exocytotic vesicle may remain close to the insertion site without collapsing and without fully releasing its cargo.

Next, we analyzed SEP-GluR1 under non-stimulated conditions and SEP-B2AR exocytosis after 30 min incubation with isoproterenol to induce recycling, and observed consecutive events similar in intensity and with similar kinetics as those observed with TfR-SEP (Figures 3C–F). Statistical analysis indicated that 12–19% of persistent events were followed by an event detected shortly at the same location (Figure 3G). As we could only count consecutive events that occurred within the 60–90 s during our imaging sessions, frequencies were most likely underestimated. Assuming that exocytosis occurs randomly at the cell surface, we determined the probability of having two consecutive events within 1 μm^2^ of each other. We developed a random model test where a threshold image of an imaged neuron was created and random events were placed on the cell surface (Figure 3H). We constrained the model such that only 10% of events for a given neuron were persistent. In addition, a co-localized event required that the first of the two events be persistent (Figure 3H). The model indicates that the probability of observing a persistent event followed by a second event is ~0.02 for a neuron with a total of 40 exocytic events (Figure 3I). Our experimental data shows that a neuron with similar surface area would present a total of ~40 events, 5–6 of which would be persistent (~10%, Figure 1J) and in average, a single persistent event would be followed by a second consecutive event. This analysis indicates that the calculated probability (~2%) is much lower than the observed frequency (>10%, Figure 3G), suggesting that consecutive events are not randomly distributed events.

**Figure 4:**
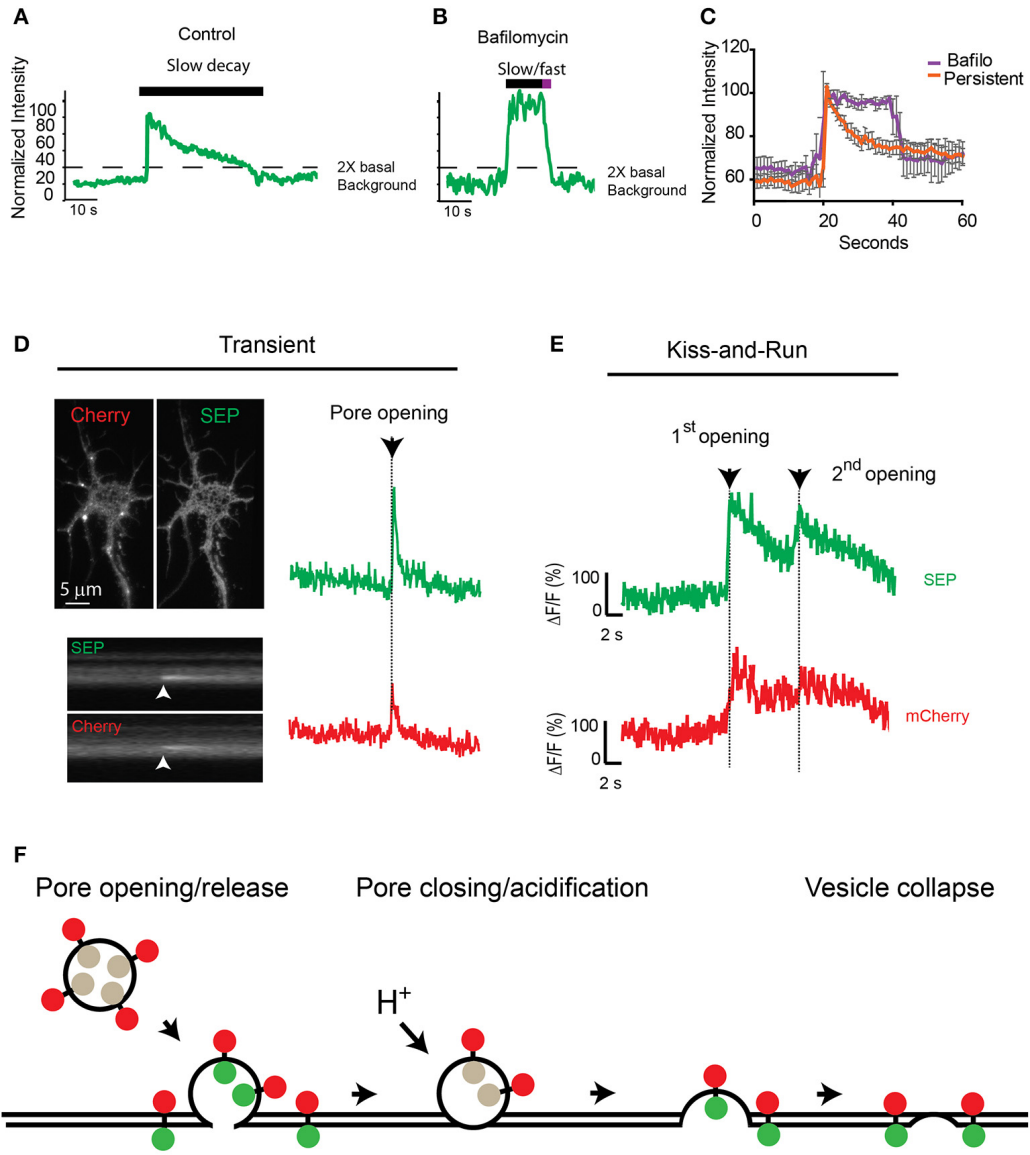
**Consecutive transferrin exocytosis is mediated by a kiss-and-run mechanism. (A)** Intensity measurements from control persistent events show slow decay kinetics (>3 s from maximum intensity to background level). **(B)** Neurons preincubated with 0.5 μM bafilomycin showed persistent events with an initial plateau followed by a fast faster decay kinetics (<3 s from maximum intensity to background levels). **(C)** Analysis of multiple persistent events from control and bafilomycin treated neurons indicate that bafilomycin created biphasic events with an initial plateau followed by a fast exponential decay (half-life ~2 s). Persistent events from control cells presented a single slow exponential decay (half-life ~17 s, see Figure 2A), errors represent standard error of the mean. **(D)** mCherry-TfR-SEP was transfected into hippocampal cultures and imaged with simultaneous dual-color TIRF. Kymographs show a transient exocytotic event in the green (SEP) and red (mCherry) channels. Maximum intensity measurements show fusion pore opening and cargo release. Arrow indicates exocytosis. **(E)** Fluorescence intensity from mCherry-TfR-SEP persistent event. Red trace (mCherry) indicates receptors are retained inside the exocytotic vesicle after an initial release to the cell surface (1st opening). Only after the second fusion pore opening, which was observed in both channels, mCherry levels reached background levels, indicating receptor release. **(F)** Exocytotic vesicles transporting mCherry-TfR-SEP are visible in the red channel (mCherry), but they are invisible in the green channel (SEP) before fusion. SEP intensity increases rapidly when the vesicular content is neutralized and mCherry-TfR-SEP is partially released onto the plasma membrane. Receptors remaining in the exocytotic vesicle are visible in the red channel. Bafilomycin prevents acidification and the decrease in SEP fluorescence.

Because fluorescence at the insertion site between consecutive events did not reach background levels (Figures 3B,D,F), we hypothesized that the fusion pore could close after an initial release of cargo and reopen for a consecutive release via a mechanism reminiscent of synaptic kiss-and-run (Stevens and Williams, 2000; Tsuboi and Rutter, 2003; Jaiswal et al., 2009; Alabi and Tsien, 2012). To test this hypothesis, we used the proton pump inhibitor bafilomycin to prevent reacidification of the closing vesicle after the initial release. Preincubation of neurons with 0.5 μM bafilomycin did not change the total proportion of TfR-SEP persistent events (control 9.8%; bafilomycin 9.3%), but bafilomycin had a significant effect on the fluorescence decay kinetics after the initial peak (Figures 4A,B). The decay kinetics, as measured from the initial maximum intensity (pore opening) to the level at which intensity was decreased two times the background levels (dashed line, Figures 4A,B), was significantly different for treated cells. Controls showed a slow one phase decay suggestive of vesicle reacidification (Figures 4A,C). In the presence of bafilomycin, an initial plateau was followed by a rapid exponential decay (Figures 4B,C). The initial plateau suggested that bafilomycin revealed receptors inside vesicles close to the plasma membrane. Complete exocytosis of these retained receptors is suggested by the fast decay observed at the end of the trace (half-life ~2 s).

Next, to visualize if TfRs are constrained at the exocytotic vesicle in a manner similar to kiss-and-run fusion, we tagged TfR-SEP with mCherry as previously utilized by Kennedy et al. (2010). These receptors will be visible at the exocytotic vesicle before and during the fusion and release process in the red channel (mCherry), whereas SEP indicates fusion pore opening and closing. mCherry-TfR-SEP expressed in HEK293 cells was visible in the red channel before fusion to the plasma membrane and during cargo release onto the plasma membrane (Supplemental Figure 2). Transient events of mCherry-TfR-SEP in hippocampal cultures were observed in both channels and were similar to those observed with TfR-SEP (Figures 1A, 4D). Persistent and consecutive events were also observed with similar frequency. During consecutive events, mCherry fluorescence intensity showed an initial increase due to the appearance of the exocytic vesicle in the evanescent field followed by a partial decrease after the first opening of the fusion pore. This first decrease indicates partial receptor release to the cell surface and reveals a pool of receptors retained at the site of insertion with little or no loss in fluorescence intensity until the second exocytotic event (Figure 4E, red trace). SEP fluorescence depicts an initial exocytic event followed by vesicle acidification and a consecutive event from the same vesicle (green trace). This result shows that after the initial release, receptors are retained inside the vesicle until a second opening of the fusion pore and subsequent release (Figures 4E,F).

## Discussion

Endosomal delivery of transmembrane receptors to the plasma membrane is fundamental in many physiological processes such as neuronal development, polarization, and synaptic plasticity (Lasiecka and Winckler, 2011). Regulated exocytosis of neurotransmitters, transmembrane proteins such as AMPA receptors, and neuropeptides has been described (Maletic-Savatic and Malinow, 1998; Maletic-Savatic et al., 1998; Wang et al., 2008; Kennedy et al., 2010; Kennedy and Ehlers, 2011). However, the mechanisms and kinetics by which exocytotic vesicles fuse and deliver transmembrane receptors to the plasma membrane remain poorly defined.

Here we investigated the vesicular fusion events mediating exocytosis of three different receptors in primary neuronal cultures. First, we showed that different receptors including TfR, a passive cargo, could be delivered to the cell surface via a persistent type of exocytosis. In our previous work, we identified the carboxyl tail of B2AR as a key element for this type of exocytosis (Yudowski et al., 2006; Yu et al., 2010). However, TfRs do not have PDZ domains such as those found in B2AR and are not bound to any known scaffold protein, suggesting that this persistent type of event is the result of a general mechanism of exocytosis and not a receptor-specific feature. Next, we characterized these persistent events by showing that they were age dependent; they did not associate with the endocytotic machinery, and their frequency nearly doubled with KCl-induced depolarization and increases in intracellular calcium as previoulsy described with synaptic vesicles and large dense-core vesicles (Klyachko and Jackson, 2002; Vardjan et al., 2007b).

A fraction of persistent events were followed by a second exocytotic event at the same location. Interestingly, we calculated that the probability of observing such consecutive events is extremely low, suggesting a regulated process. Finally, we double tagged TfR with mCherry and SEP to simultaneously image cargo release and its retention in exocytic vesicles. Dual-color TIRF imaging showed that after initial release, the fusion pore closed and reopened for a subsequent delivery similar to the fusion pore flickering observed during kiss-and-run and as recently reported by others (Jullié et al., 2014). The kinetics of reacidification are not constant for all vesicles and are much slower than synaptic reacidification (Budzinski et al., 2011). One explanation is that synaptic vesicles are significantly smaller than endosomal vesicles and their acidification occurs much more quickly (Sudhof, 2004). Another possibility is that these exocytotic events are not mediated by discrete vesicles but by recycling tubules connected to endosomes, effectively increasing their area and slowing their acidification (Puthenveedu et al., 2010; Jullié et al., 2014).

Neurotransmitter and peptide release by kiss-and-run has been extensively described before (Staal et al., 2004; Vardjan et al., 2007a). During these events, the fusion pore open and closes multiple times, selectively controlling cargo delivery (Ryan, 2003; Staal et al., 2004; Vardjan et al., 2007a, b, 2013). Our results together with Perrais et al. (Jullié et al., 2014) suggest kiss-and-run as a general mechanism for the delivery of transmembrane receptors to plasma membrane. Transient and persistent events can co-exist to dynamically and precisely control the delivery of different types of cargo to the surface. These exocytotic mechanisms may contribute to the rapid and specific changes observed in receptor levels during depolarization. Further studies will be needed to define the mechanisms controlling fusion pore stability and receptor release by kiss-and-run.

## Methods

### cDNA constructs

SEP-B2AR, SEP-MOR, SEP-GluR1, and TfR-SEP have been previously described (Yudowski et al., 2006, 2007, 2009; Yu et al., 2010). To produce mCherry-TfR-SEP, mCherry was amplified with PCR using primers that include Agel flanking sites: 5'-GCGCGCGCGCACCGGTAGTGAGCAAGGGCGAGGAGGATAACA-3' and 5'-GCGCGCGCGCACCGGTATCTTGTACAGCTCGTCCATGCCG-3'. The fragment was then cloned into TfR-SEP. Clathrin light chain DsRed (CLC-DsRed) was a gift from Wolf Almers (Vollum Institute, Oregon). All chemicals were purchased from Sigma-Aldrich except dyngo-4a, which was purchased from Abcam (Cambridge, MA).

### Hippocampal and striatal cultures

Dissociated primary cultures were either dissected from embryonic day 17–18 Sprague-Dawley rat embryos of either sex (Yu et al., 2010) or purchased from BrainBits LLC (Springfield, IL). Dissected tissue was dissociated in 1× trypsin/EDTA solution (Invitrogen, Gaithersburg, MD) for 15 min, and then 1 mL of trypsin inhibitor was added for 5 min at room temperature. Cells were washed and triturated in Dulbecco's modified Eagle's medium containing 10% fetal calf serum (Invitrogen) using a glass pipette. Neurons were transfected with 2–3 μg cDNA per 300,000 neurons after 5–7 DIV using Lipofectamine 2000 (Invitrogen) according to the manufacturer's protocol. Hippocampal cultures were ~90% pure as calculated by MAP2 and GFAP staining as previously described (Yudowski et al., 2007).

### Live-cell TIRF imaging

Imaging was performed using a fully motorized Nikon (Melville, NY) Ti-E inverted microscope with a CFI-APO 100×, 1.49 numerical aperture TIRF objective and a motorized stage. Coherent sapphire lasers (488 and 561 nm; Coherent Inc., Santa Clara, CA) were used as a light source for TIRF microscopy illumination modes. The laser was focused using the back focal plane before each experiment. Images were acquired at 10 Hz using an iXonEM+ DU897 back-illuminated EMCCD camera (Andor, Belfast, UK), which was operated in the linear range during all imaging sessions. The sample temperature was controlled at 37°C using a Bioptechs Stable Z stage or objective warmer (Bioptechs, Butler, PA). Simultaneous dual-color images were acquired with a dual-view (Photometrics, Tucson, AZ). Images shown represent raw data with simple background subtraction of the averaged blank field intensity. Intensity values were corrected for photobleaching during the image collection (usually 2–10% from initial measurements; see Supplemental Figure 1). Treatments and washes were performed by perfusion. Imaging was performed in neurons from DIV 7–25. Neurons were first identified under epifluorescence illumination, and then the illumination was switched to TIRF (27). Neurons were first preincubated for 20–30 min in imaging media (Opti-MEM® reduced-serum medium (Invitrogen) supplemented with 20 mM HEPES (Invitrogen). Depolarization was achieved by perfusion with 25 mM KCL. 1 μM A23187 was present in the imaging media during calcium experiments.

### Statistical and probabilistic analysis of exocytotic events

Exocytotic events were identified and counted blindly as described previously, and images were background subtracted (Yudowski et al., 2007, 2009). Analysis was performed using the public domain NIH Image program ImageJ/FIJI software, which is freely available at http://fiji.sc/Fiji. To analyze statistical significance between groups, we counted the number of events in each independent experiment (i.e., each separate imaging session and different dishes of cultured neurons were treated as independent experiments), analyzed their normality by D'Agostino and Pearson test and used unpaired two-tailed Student's *t*-tests to test for statistical significance. We used GraphPad Prism software (San Diego, CA) with a significance threshold of *p* < 0.05. Standard deviations are shown unless indicated and ImageJ to obtain Pearson's correlation coefficient. Exponential decay and half-lives values were obtained by applying a plateau followed by one phase decay kinetic equation.

The probability of consecutive events was obtained by generating a thresholded image [*It*_(*i,j*)_ = 1, if *I*_(*i,j*)_ ≥ *T*, otherwise *It*_(*i,j*)_ = 0] of the neuron to approximate the projected image. Here [*i* and *j* OR *i;j* OR *i,j*] are the *x*–*y* pixel coordinates. A given numerical experiment places *N* events randomly on pixels whose *It*_(*i,j*)_ = 1 (e.g., red squares in the thresholded image in Figure 3G with 15,318 pixels in the thresholded neuron).

### Conflict of interest statement

The authors declare that the research was conducted in the absence of any commercial or financial relationships that could be construed as a potential conflict of interest.

## Supplementary material

Supplemental Figure 1 **Intensity measurements over time from a hippocampal neuron transfected with TfR-SEP**. Changes in total fluorescence intensity from the cell surface (bleaching) were compared with the intensity changes from a persistent event (event). Fluorescence was analyzed in an identical region of interest on the same cell. (Average bleaching after 1 min = 5–7%; *n* = 8 cells.)

Supplemental Figure 2 **HEK293 cells were transfected with mCherry-TfR-SEP and imaged with TIRF microscopy. (A)** Sequence and kymographs from simultaneous dual-color imaging showing a transient event. **(B)** Fluorescence traces from the event in **(A)** indicating vesicle docking, pore opening, and cargo release. **(C)** Cartoon depicting vesicle fluorescence before and after pore opening.

Supplemental Movie 1 **Dissociated hippocampal neuron (>15 DIV) transfected with TfR-SEP and imaged under TIRF illumination at 10 Hz**. Individual exocytic events are visible as rapid increases in surface fluorescence.
