# Supplementary figures and images for: Imaging of kiss-and-run exocytosis of surface receptors in neuronal cultures
Source: Front Cell Neurosci. 2014 Nov 3;8:363. doi: 10.3389/fncel.2014.00363 (PMC4217495; doi:10.3389/fncel.2014.00363)

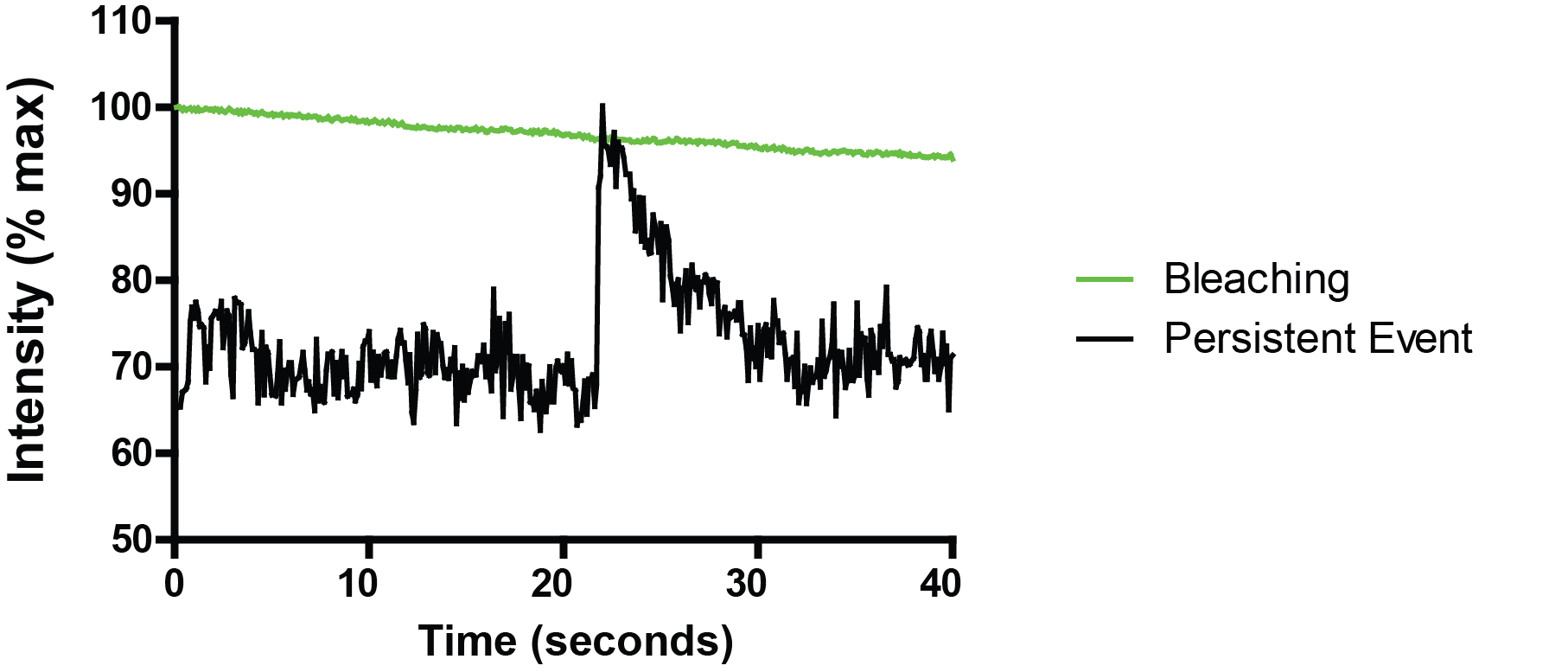

Supplement: Supplemental Figure 1 — Intensity measurements over time from a hippocampal neuron transfected with TfR-SEP. Changes in total fluorescence intensity from the cell surface (bleaching) were compared with the intensity changes from a persistent event (event). Fluorescence was analyzed in an identical region of interest on the same cell. (Average bleaching after 1 min = 5–7%; n = 8 cells.) [file Image1.TIF]

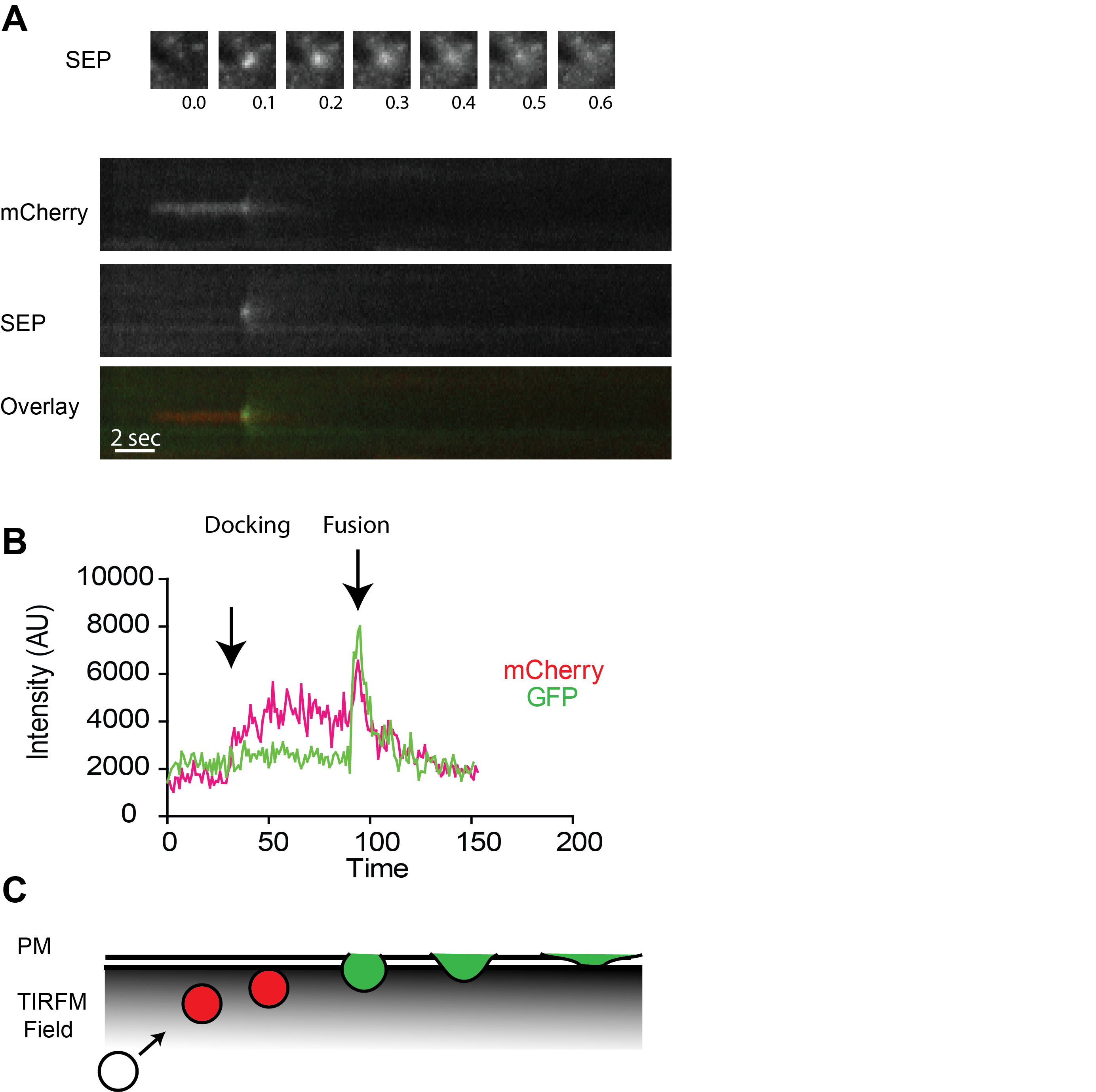

Supplement: Supplemental Figure 2 — HEK293 cells were transfected with mCherry-TfR-SEP and imaged with TIRF microscopy. (A) Sequence and kymographs from simultaneous dual-color imaging showing a transient event. (B) Fluorescence traces from the event in (A) indicating vesicle docking, pore opening, and cargo release. (C) Cartoon depicting vesicle fluorescence before and after pore opening. [file Image2.TIF]
